# Supplementary material for: Drought adaptability of different subspecies of tetraploid wheat (Triticum turgidum) under contrasting moisture conditions: Association with solvent retention capacity and quality-related traits
Source: PLoS One. 2023 Feb 7;18(2):e0275412. doi: 10.1371/journal.pone.0275412 (PMC10045581; doi:10.1371/journal.pone.0275412)
Supplement: S1 Table — (DOC) [file pone.0275412.s001.doc]

| **S1 Table -** Mean comparisons of different traits evaluated on 36 genotypes of *wheat* during two years (2019 and 2020) under well-watering (WW) and water stress (WS) conditions. | | | | | | | | | | | | | | | | | | | | | | | | | | | | | | | | | | | | | | | | | | | | |
| --- | --- | --- | --- | --- | --- | --- | --- | --- | --- | --- | --- | --- | --- | --- | --- | --- | --- | --- | --- | --- | --- | --- | --- | --- | --- | --- | --- | --- | --- | --- | --- | --- | --- | --- | --- | --- | --- | --- | --- | --- | --- | --- | --- | --- |
| Traits | Thousand grain weight (g) | | | | | | | | | |  | | Grain yield (g/m^2^) | | | | | | | | |  | Percentage of grain protein (%) | | | | | | | | | | | |  | Zeleny index (%) | | | | | | | | |
| Genotype | 2019 | | | |  | | 2020 | | | |  | | 2019 | | | |  | | 2020 | | |  | 2019 | | | | |  | | 2020 | | | | |  | 2019 | | | |  | | 2020 | | |
|  | WW | | WS | |  | | WW | | WS | |  | | WW | | WS | |  | | WW | | WS |  | WW | | | WS | |  | | WW | | WS | | |  | WW | | WS | |  | | WW | | WS |
| G1 | 39.78 | | 46.18 | |  | | 41.48 | | 32.53 | |  | | 718.41 | | 668.83 | |  | | 730.00 | | 654.05 |  | 12.31 | | | 12.83 | |  | | 11.67 | | 12.65 | | |  | 31.15 | | 32.34 | |  | | 28.22 | | 31.65 |
| G2 | 46.06 | | 43.26 | |  | | 45.12 | | 37.58 | |  | | 822.81 | | 760.34 | |  | | 813.28 | | 828.97 |  | 12.10 | | | 13.67 | |  | | 11.08 | | 12.17 | | |  | 31.03 | | 33.46 | |  | | 29.22 | | 33.28 |
| G3 | 51.37 | | 43.40 | |  | | 38.65 | | 34.28 | |  | | 686.49 | | 572.41 | |  | | 718.04 | | 485.43 |  | 13.78 | | | 14.80 | |  | | 11.73 | | 13.26 | | |  | 32.27 | | 32.59 | |  | | 29.28 | | 34.65 |
| G4 | 52.25 | | 45.25 | |  | | 40.82 | | 35.97 | |  | | 761.11 | | 632.00 | |  | | 686.19 | | 634.58 |  | 12.96 | | | 13.42 | |  | | 11.36 | | 12.53 | | |  | 31.59 | | 32.65 | |  | | 27.59 | | 36.40 |
| G5 | 44.51 | | 41.30 | |  | | 40.40 | | 32.47 | |  | | 783.84 | | 668.26 | |  | | 817.89 | | 674.05 |  | 12.65 | | | 13.02 | |  | | 11.52 | | 13.56 | | |  | 31.15 | | 32.40 | |  | | 33.84 | | 43.53 |
| G6 | 53.54 | | 49.15 | |  | | 44.82 | | 36.22 | |  | | 760.60 | | 629.44 | |  | | 918.60 | | 631.17 |  | 13.22 | | | 15.17 | |  | | 11.86 | | 13.02 | | |  | 32.71 | | 35.39 | |  | | 35.65 | | 31.96 |
| G7 | 46.28 | | 35.59 | |  | | 32.77 | | 24.63 | |  | | 766.85 | | 456.04 | |  | | 729.69 | | 381.85 |  | 12.29 | | | 12.65 | |  | | 11.88 | | 12.04 | | |  | 31.53 | | 31.71 | |  | | 24.66 | | 28.47 |
| G8 | 39.39 | | 43.20 | |  | | 37.67 | | 32.35 | |  | | 632.56 | | 560.57 | |  | | 784.86 | | 532.03 |  | 13.49 | | | 14.16 | |  | | 11.62 | | 13.05 | | |  | 31.84 | | 33.52 | |  | | 29.28 | | 30.96 |
| G9 | 33.21 | | 28.01 | |  | | 29.25 | | 30.80 | |  | | 450.15 | | 396.26 | |  | | 410.06 | | 441.70 |  | 14.36 | | | 16.22 | |  | | 13.71 | | 13.93 | | |  | 31.84 | | 33.71 | |  | | 35.15 | | 30.96 |
| G10 | 31.17 | | 27.91 | |  | | 30.18 | | 33.48 | |  | | 432.24 | | 370.44 | |  | | 442.33 | | 321.43 |  | 14.57 | | | 15.48 | |  | | 13.42 | | 15.59 | | |  | 31.78 | | 33.46 | |  | | 26.28 | | 32.84 |
| G11 | 32.35 | | 28.19 | |  | | 29.12 | | 31.07 | |  | | 395.86 | | 345.94 | |  | | 411.68 | | 304.04 |  | 15.78 | | | 16.30 | |  | | 13.53 | | 15.81 | | |  | 33.52 | | 33.96 | |  | | 29.84 | | 33.90 |
| G12 | 33.50 | | 26.76 | |  | | 31.13 | | 32.58 | |  | | 470.35 | | 390.09 | |  | | 489.49 | | 350.05 |  | 14.25 | | | 14.90 | |  | | 13.33 | | 14.61 | | |  | 31.90 | | 32.84 | |  | | 31.03 | | 32.03 |
| G13 | 38.24 | | 31.42 | |  | | 34.82 | | 33.58 | |  | | 667.18 | | 313.13 | |  | | 597.53 | | 353.98 |  | 11.78 | | | 12.54 | |  | | 11.15 | | 12.15 | | |  | 31.15 | | 32.15 | |  | | 37.84 | | 31.71 |
| G14 | 33.53 | | 32.70 | |  | | 35.67 | | 29.20 | |  | | 375.43 | | 328.05 | |  | | 382.13 | | 342.70 |  | 13.89 | | | 15.39 | |  | | 13.68 | | 13.20 | | |  | 32.90 | | 34.21 | |  | | 31.09 | | 31.15 |
| G15 | 37.79 | | 33.55 | |  | | 31.85 | | 28.07 | |  | | 395.33 | | 371.23 | |  | | 432.78 | | 452.01 |  | 14.48 | | | 15.40 | |  | | 14.25 | | 16.45 | | |  | 33.52 | | 34.46 | |  | | 34.40 | | 36.46 |
| G16 | 36.64 | | 32.55 | |  | | 30.80 | | 31.30 | |  | | 403.49 | | 357.93 | |  | | 457.19 | | 368.76 |  | 14.16 | | | 16.71 | |  | | 14.65 | | 15.39 | | |  | 33.52 | | 36.14 | |  | | 34.71 | | 35.89 |
| G17 | 36.75 | | 30.76 | |  | | 29.25 | | 32.20 | |  | | 400.01 | | 378.18 | |  | | 387.27 | | 378.29 |  | 14.86 | | | 15.13 | |  | | 14.10 | | 16.50 | | |  | 34.08 | | 34.02 | |  | | 34.27 | | 36.89 |
| G18 | 45.78 | | 44.75 | |  | | 55.12 | | 47.03 | |  | | 514.61 | | 462.18 | |  | | 449.50 | | 412.52 |  | 13.06 | | | 13.67 | |  | | 11.44 | | 12.49 | | |  | 31.21 | | 32.84 | |  | | 35.53 | | 30.34 |
| G19 | 47.60 | | 39.36 | |  | | 48.42 | | 43.80 | |  | | 496.41 | | 281.25 | |  | | 494.59 | | 221.78 |  | 13.64 | | | 15.67 | |  | | 12.45 | | 14.25 | | |  | 34.21 | | 34.77 | |  | | 32.09 | | 33.02 |
| G20 | 35.35 | | 34.97 | |  | | 29.30 | | 33.73 | |  | | 444.91 | | 361.31 | |  | | 424.56 | | 346.45 |  | 13.43 | | | 14.57 | |  | | 12.18 | | 13.62 | | |  | 32.09 | | 32.65 | |  | | 36.47 | | 32.27 |
| G21 | 54.58 | | 48.99 | |  | | 48.95 | | 44.38 | |  | | 591.69 | | 483.79 | |  | | 620.98 | | 413.55 |  | 14.14 | | | 14.16 | |  | | 13.32 | | 12.89 | | |  | 33.59 | | 33.21 | |  | | 32.84 | | 30.96 |
| G22 | 52.92 | | 45.39 | |  | | 46.22 | | 44.95 | |  | | 417.83 | | 313.84 | |  | | 400.29 | | 303.78 |  | 14.65 | | | 15.86 | |  | | 13.00 | | 13.97 | | |  | 34.90 | | 36.27 | |  | | 32.96 | | 33.90 |
| G23 | 36.79 | | 30.89 | |  | | 35.38 | | 29.87 | |  | | 675.48 | | 430.53 | |  | | 708.92 | | 448.44 |  | 15.63 | | | 16.97 | |  | | 15.12 | | 15.26 | | |  | 35.39 | | 36.58 | |  | | 36.39 | | 35.64 |
| G24 | 27.91 | | 27.43 | |  | | 39.78 | | 29.63 | |  | | 474.55 | | 317.61 | |  | | 397.23 | | 281.26 |  | 13.86 | | | 15.49 | |  | | 13.82 | | 14.38 | | |  | 32.65 | | 35.27 | |  | | 32.21 | | 33.65 |
| G25 | 31.66 | | 27.56 | |  | | 26.02 | | 25.85 | |  | | 310.87 | | 116.22 | |  | | 286.59 | | 113.89 |  | 17.07 | | | 14.99 | |  | | 15.78 | | 16.65 | | |  | 34.15 | | 32.77 | |  | | 32.03 | | 32.77 |
| G26 | 37.83 | | 31.50 | |  | | 31.63 | | 26.12 | |  | | 536.27 | | 469.50 | |  | | 464.19 | | 595.18 |  | 13.32 | | | 14.95 | |  | | 12.69 | | 14.44 | | |  | 33.21 | | 34.15 | |  | | 36.21 | | 31.15 |
| G27 | 48.99 | | 43.66 | |  | | 46.77 | | 48.23 | |  | | 595.80 | | 529.29 | |  | | 531.79 | | 481.26 |  | 14.65 | | | 16.08 | |  | | 11.89 | | 13.36 | | |  | 33.65 | | 35.15 | |  | | 32.47 | | 32.46 |
| G28 | 47.20 | | 42.29 | |  | | 46.33 | | 28.98 | |  | | 763.28 | | 569.29 | |  | | 827.10 | | 542.94 |  | 12.42 | | | 13.19 | |  | | 11.42 | | 12.68 | | |  | 32.09 | | 32.65 | |  | | 41.28 | | 31.40 |
| G29 | 34.94 | | 29.47 | |  | | 37.55 | | 39.72 | |  | | 481.14 | | 253.80 | |  | | 433.63 | | 214.91 |  | 14.40 | | | 15.22 | |  | | 13.36 | | 16.05 | | |  | 34.33 | | 33.52 | |  | | 30.96 | | 32.90 |
| G30 | 35.28 | | 26.96 | |  | | 35.93 | | 35.53 | |  | | 214.69 | | 82.96 | |  | | 236.14 | | 57.25 |  | 14.91 | | | 16.50 | |  | | 13.54 | | 13.70 | | |  | 33.90 | | 34.90 | |  | | 33.02 | | 32.96 |
| G31 | 28.60 | | 26.24 | |  | | 28.88 | | 30.27 | |  | | 288.72 | | 191.68 | |  | | 259.44 | | 158.16 |  | 13.53 | | | 15.36 | |  | | 12.71 | | 14.86 | | |  | 31.84 | | 33.46 | |  | | 31.21 | | 33.90 |
| G32 | 38.79 | | 30.84 | |  | | 30.77 | | 31.98 | |  | | 488.46 | | 262.10 | |  | | 646.57 | | 237.57 |  | 12.33 | | | 15.20 | |  | | 11.81 | | 13.37 | | |  | 31.40 | | 33.96 | |  | | 35.53 | | 32.52 |
| G33 | 51.41 | | 40.41 | |  | | 36.75 | | 36.65 | |  | | 515.01 | | 332.65 | |  | | 610.22 | | 250.24 |  | 11.51 | | | 14.09 | |  | | 11.31 | | 12.26 | | |  | 30.09 | | 33.21 | |  | | 38.40 | | 31.09 |
| G34 | 47.80 | | 41.85 | |  | | 33.60 | | 25.83 | |  | | 669.14 | | 628.33 | |  | | 691.54 | | 637.02 |  | 11.31 | | | 11.81 | |  | | 10.81 | | 11.79 | | |  | 29.78 | | 32.65 | |  | | 34.40 | | 31.21 |
| G35 | 39.42 | | 37.77 | |  | | 28.00 | | 28.57 | |  | | 550.88 | | 503.26 | |  | | 608.47 | | 577.23 |  | 12.56 | | | 13.49 | |  | | 12.10 | | 11.94 | | |  | 31.90 | | 33.15 | |  | | 34.90 | | 35.03 |
| G36 | 48.87 | | 40.89 | |  | | 36.32 | | 37.53 | |  | | 571.47 | | 429.19 | |  | | 553.90 | | 350.60 |  | 13.82 | | | 14.24 | |  | | 12.74 | | 13.00 | | |  | 32.59 | | 32.46 | |  | | 31.65 | | 32.03 |
| LSD_E_(α = 0.05) | 4.07 | | 4.72 | |  | | 6.89 | | 6.35 | |  | | 5.79 | | 6.60 | |  | | 10.81 | | 10.06 |  | 227.13 | | | 193.78 | |  | | 253.67 | | 227.94 | | |  | 0.81 | | 0.83 | |  | | 0.93 | | 0.90 |
| LSD_E×Y_ (α=0.05) | 3.06 | | | |  | | 4.60 | | | |  | | 4.31 | | | |  | | 7.25 | | |  | 146.66 | | | | |  | | 167.52 | | | | |  | 0.57 | | | |  | | 0.64 | | |
| **S1 Table -** Continued | | | | | | | | | | | | | | | | | | | | | | | | | | | | | | | | | | | | | | | | | | | | |
| Traits | | Grain hardiness (%) | | | | | | | |  | | Moisture of flour (%) | | | | | | | | | |  | | Water absorption of flour (%) | | | | | | | | |  | Zn (mg/g) | | | | | | | | | | |
| Genotype | | 2019 | |  | | 2020 | | | |  | | 2019 | | | |  | | 2020 | | | |  | | 2019 | | |  | | 2020 | | | |  | 2019 | | | | |  | | 2020 | | | |
|  |  | WW | WS |  | | WW | | WS | |  | | WW | | WS | |  | | WW | | WS | |  | | WW | WS | |  | | WW | | WS | |  | WW | | | WS | |  | | WW | | WS | |
| G1 | | 69.00 | 70.50 |  | | 59.50 | | 59.50 | |  | | 8.45 | | 8.40 | |  | | 11.85 | | 11.45 | |  | | 68.90 | 69.55 | |  | | 66.65 | | 67.65 | |  | 0.57 | | | 0.53 | |  | | 0.44 | | 0.69 | |
| G2 | | 68.00 | 73.50 |  | | 61.50 | | 57.00 | |  | | 8.65 | | 7.70 | |  | | 12.25 | | 12.05 | |  | | 68.80 | 69.90 | |  | | 67.00 | | 67.10 | |  | 0.59 | | | 0.55 | |  | | 0.28 | | 0.61 | |
| G3 | | 68.00 | 70.00 |  | | 61.50 | | 62.50 | |  | | 8.40 | | 7.90 | |  | | 12.40 | | 11.70 | |  | | 68.80 | 69.55 | |  | | 67.20 | | 67.85 | |  | 0.61 | | | 0.63 | |  | | 0.50 | | 0.53 | |
| G4 | | 64.50 | 71.00 |  | | 58.50 | | 62.50 | |  | | 8.50 | | 8.00 | |  | | 12.30 | | 11.95 | |  | | 68.15 | 69.80 | |  | | 66.35 | | 68.15 | |  | 0.63 | | | 0.59 | |  | | 0.48 | | 0.52 | |
| G5 | | 68.00 | 72.50 |  | | 63.00 | | 57.00 | |  | | 8.25 | | 8.05 | |  | | 12.20 | | 11.30 | |  | | 68.30 | 69.85 | |  | | 67.65 | | 66.90 | |  | 0.59 | | | 0.55 | |  | | 0.52 | | 0.69 | |
| G6 | | 70.50 | 76.50 |  | | 64.00 | | 64.50 | |  | | 8.30 | | 7.45 | |  | | 11.95 | | 11.65 | |  | | 69.40 | 71.10 | |  | | 68.15 | | 68.50 | |  | 0.58 | | | 0.51 | |  | | 0.54 | | 0.58 | |
| G7 | | 70.00 | 72.50 |  | | 54.50 | | 54.50 | |  | | 8.70 | | 7.90 | |  | | 12.00 | | 12.30 | |  | | 69.70 | 69.70 | |  | | 65.60 | | 65.35 | |  | 0.57 | | | 0.50 | |  | | 0.58 | | 0.66 | |
| G8 | | 68.50 | 78.00 |  | | 62.00 | | 65.00 | |  | | 8.70 | | 7.55 | |  | | 12.45 | | 12.20 | |  | | 69.65 | 71.15 | |  | | 67.30 | | 68.60 | |  | 0.59 | | | 0.55 | |  | | 0.49 | | 0.60 | |
| G9 | | 66.00 | 71.50 |  | | 63.00 | | 61.50 | |  | | 9.15 | | 8.30 | |  | | 12.05 | | 12.05 | |  | | 69.70 | 71.35 | |  | | 69.15 | | 68.75 | |  | 0.59 | | | 0.68 | |  | | 0.56 | | 0.66 | |
| G10 | | 66.00 | 73.00 |  | | 58.50 | | 62.00 | |  | | 9.10 | | 8.75 | |  | | 12.75 | | 11.70 | |  | | 69.70 | 72.05 | |  | | 67.45 | | 69.90 | |  | 0.68 | | | 0.69 | |  | | 0.49 | | 0.77 | |
| G11 | | 71.50 | 73.50 |  | | 57.00 | | 66.50 | |  | | 8.65 | | 8.40 | |  | | 12.65 | | 10.90 | |  | | 71.25 | 71.70 | |  | | 67.55 | | 70.20 | |  | 0.67 | | | 0.57 | |  | | 0.59 | | 0.78 | |
| G12 | | 66.50 | 71.00 |  | | 58.00 | | 64.00 | |  | | 9.10 | | 8.80 | |  | | 12.10 | | 11.75 | |  | | 69.80 | 71.50 | |  | | 67.20 | | 69.95 | |  | 0.68 | | | 0.63 | |  | | 0.56 | | 0.64 | |
| G13 | | 49.50 | 50.50 |  | | 42.50 | | 43.50 | |  | | 9.00 | | 8.35 | |  | | 12.10 | | 11.50 | |  | | 63.80 | 64.25 | |  | | 62.15 | | 62.90 | |  | 0.53 | | | 0.52 | |  | | 0.46 | | 0.60 | |
| G14 | | 64.50 | 72.50 |  | | 61.00 | | 56.00 | |  | | 9.50 | | 8.15 | |  | | 12.20 | | 11.85 | |  | | 69.65 | 71.05 | |  | | 68.85 | | 67.40 | |  | 0.66 | | | 0.61 | |  | | 0.58 | | 0.74 | |
| G15 | | 60.00 | 64.00 |  | | 56.00 | | 60.50 | |  | | 8.30 | | 8.05 | |  | | 11.40 | | 10.90 | |  | | 66.55 | 67.65 | |  | | 65.95 | | 67.50 | |  | 0.59 | | | 0.52 | |  | | 0.54 | | 0.73 | |
| G16 | | 60.00 | 67.00 |  | | 55.50 | | 60.00 | |  | | 8.65 | | 7.70 | |  | | 11.30 | | 10.55 | |  | | 66.80 | 68.65 | |  | | 66.00 | | 67.10 | |  | 0.60 | | | 0.63 | |  | | 0.48 | | 0.61 | |
| G17 | | 61.00 | 65.50 |  | | 56.50 | | 59.00 | |  | | 8.50 | | 7.80 | |  | | 11.45 | | 10.75 | |  | | 67.20 | 68.05 | |  | | 66.05 | | 67.30 | |  | 0.63 | | | - | |  | | 0.47 | | 0.65 | |
| G18 | | 65.50 | 74.50 |  | | 62.50 | | 62.00 | |  | | 8.60 | | 7.70 | |  | | 11.80 | | 11.65 | |  | | 68.50 | 70.75 | |  | | 67.00 | | 68.10 | |  | 0.61 | | | 0.48 | |  | | 0.51 | | 0.56 | |
| G19 | | 70.00 | 73.50 |  | | 63.00 | | 61.00 | |  | | 8.65 | | 7.85 | |  | | 11.75 | | 11.30 | |  | | 70.70 | 71.45 | |  | | 68.25 | | 68.25 | |  | 0.62 | | | 0.56 | |  | | 0.56 | | 0.71 | |
| G20 | | 71.50 | 74.00 |  | | 63.50 | | 64.00 | |  | | 8.10 | | 7.65 | |  | | 12.45 | | 11.25 | |  | | 69.55 | 70.30 | |  | | 68.55 | | 69.10 | |  | 0.60 | | | 0.63 | |  | | 0.54 | | 0.73 | |
| G21 | | 36.50 | 70.00 |  | | 68.00 | | 57.50 | |  | | 8.60 | | 8.00 | |  | | 12.10 | | 12.00 | |  | | 69.45 | 69.90 | |  | | 70.05 | | 67.50 | |  | 0.67 | | | 0.65 | |  | | 0.59 | | 0.64 | |
| G22 | | 69.50 | 76.00 |  | | 65.50 | | 60.00 | |  | | 8.55 | | 8.00 | |  | | 11.85 | | 11.80 | |  | | 70.45 | 72.40 | |  | | 69.35 | | 69.15 | |  | 0.71 | | | 0.64 | |  | | 0.66 | | 0.81 | |
| G23 | | 61.50 | 68.50 |  | | 63.50 | | 60.00 | |  | | 8.35 | | 7.75 | |  | | 11.85 | | 11.45 | |  | | 67.90 | 69.80 | |  | | 68.90 | | 67.65 | |  | 0.66 | | | 0.68 | |  | | 0.59 | | 0.55 | |
| G24 | | 63.00 | 72.50 |  | | 60.50 | | 59.50 | |  | | 9.00 | | 7.90 | |  | | 11.75 | | 11.80 | |  | | 68.75 | 71.05 | |  | | 68.20 | | 68.40 | |  | 0.69 | | | 0.65 | |  | | 0.64 | | 0.61 | |
| G25 | | 69.00 | 62.00 |  | | 63.50 | | 60.50 | |  | | 8.90 | | 9.85 | |  | | 12.20 | | 11.20 | |  | | 70.35 | 67.40 | |  | | 69.70 | | 68.95 | |  | 0.77 | | | - | |  | | 0.75 | | 0.84 | |
| G26 | | 72.00 | 75.50 |  | | 66.00 | | 57.50 | |  | | 8.35 | | 7.55 | |  | | 12.00 | | 11.85 | |  | | 70.25 | 70.80 | |  | | 68.65 | | 67.30 | |  | 0.65 | | | 0.59 | |  | | 0.60 | | 0.68 | |
| G27 | | 70.00 | 76.50 |  | | 64.00 | | 67.00 | |  | | 8.40 | | 7.20 | |  | | 12.15 | | 11.50 | |  | | 70.30 | 71.25 | |  | | 68.00 | | 68.95 | |  | 0.73 | | | 0.67 | |  | | 0.57 | | 0.55 | |
| G28 | | 61.50 | 63.50 |  | | 50.50 | | 50.50 | |  | | 8.30 | | 7.70 | |  | | 12.10 | | 11.55 | |  | | 66.60 | 66.90 | |  | | 64.45 | | 64.85 | |  | 0.57 | | | 0.54 | |  | | 0.50 | | 0.60 | |
| G29 | | 73.00 | 67.50 |  | | 61.00 | | 61.50 | |  | | 9.05 | | 9.00 | |  | | 12.25 | | 11.50 | |  | | 71.40 | 70.15 | |  | | 67.55 | | 66.95 | |  | 0.69 | | | 0.69 | |  | | 0.64 | | 0.96 | |
| G30 | | 69.50 | 67.00 |  | | 64.50 | | 64.50 | |  | | 8.50 | | 8.70 | |  | | 11.65 | | 11.50 | |  | | 70.65 | 70.70 | |  | | 69.00 | | 69.25 | |  | 0.81 | | | 0.83 | |  | | 0.75 | | 0.74 | |
| G31 | | 63.50 | 64.50 |  | | 64.00 | | 63.50 | |  | | 9.25 | | 8.60 | |  | | 12.05 | | 11.00 | |  | | 69.00 | 69.20 | |  | | 68.00 | | 69.45 | |  | 0.62 | | | 0.74 | |  | | - | | 0.66 | |
| G32 | | 68.00 | 73.50 |  | | 61.50 | | 63.00 | |  | | 8.35 | | 8.35 | |  | | 11.65 | | 11.75 | |  | | 68.10 | 70.65 | |  | | 66.60 | | 68.95 | |  | 0.59 | | | 0.67 | |  | | - | | 0.68 | |
| G33 | | 50.50 | 68.00 |  | | 52.50 | | 48.00 | |  | | 9.05 | | 8.15 | |  | | 12.55 | | 11.55 | |  | | 63.90 | 69.40 | |  | | 65.45 | | 64.65 | |  | 0.57 | | | 0.70 | |  | | - | | 0.64 | |
| G34 | | 48.00 | 55.00 |  | | 41.00 | | 41.00 | |  | | 8.85 | | 8.40 | |  | | 11.80 | | 11.30 | |  | | 63.35 | 65.10 | |  | | 60.80 | | 61.75 | |  | 0.72 | | | 0.45 | |  | | - | | 0.69 | |
| G35 | | 56.00 | 58.00 |  | | 55.00 | | 50.50 | |  | | 8.65 | | 8.05 | |  | | 11.65 | | 11.75 | |  | | 65.45 | 66.20 | |  | | 65.40 | | 64.65 | |  | 0.69 | | | 0.48 | |  | | - | | 0.50 | |
| G36 | | 70.00 | 65.50 |  | | 63.00 | | 65.50 | |  | | 8.35 | | 8.40 | |  | | 12.25 | | 12.20 | |  | | 69.70 | 69.05 | |  | | 67.90 | | 68.80 | |  | 0.57 | | | 0.59 | |  | | 0.54 | | 0.54 | |
| LSD_E_(α = 0.05) | | 14.83 | 5.64 |  | | 4.28 | | 4.93 | |  | | 0.63 | | 0.55 | |  | | 0.49 | | 0.47 | |  | | 1.18 | 1.69 | |  | | 1.32 | | 1.48 | |  | 0.08 | | | 0.09 | |  | | 0.12 | | 0.18 | |
| LSD_E×Y_ (α=0.05) | | 7.79 | |  | | 3.21 | | | |  | | 0.41 | | | |  | | 0.33 | | | |  | | 1.01 | | |  | | 0.97 | | | |  | 0.08 | | | | |  | | 0.15 | | | |
| **S1 Table -** Continued | | | | | | | | | | | | | | | | | | | | | | | | | | | | | | | | | | | | | | | | | | | | |
| Traits | | Fe (mg/g) | | | | | | | |  | | Na^+^ (mg/g) | | | | | | | | | |  | | K^+^ (mg/g) | | | | | | | | |  | K^+^ / Na^+^ | | | | | | | | | | |
| Genotype | | 2019 | |  | | 2020 | | | |  | | 2019 | | | |  | | 2020 | | | |  | | 2019 | | |  | | 2020 | | | |  | 2019 | | | | |  | | 2020 | | | |
|  |  | WW | WS |  | | WW | | WS | |  | | WW | | WS | |  | | WW | | WS | |  | | WW | WS | |  | | WW | | WS | |  | WW | | | WS | |  | | WW | | WS | |
| G1 | | 0.45 | 0.52 |  | | 0.33 | | 0.56 | |  | | 4.43 | | 4.57 | |  | | 4.43 | | 6.63 | |  | | 7.86 | 7.38 | |  | | 6.98 | | 9.69 | |  | 1.78 | | | 1.62 | |  | | 1.58 | | 1.48 | |
| G2 | | 0.42 | 0.50 |  | | 0.21 | | 0.54 | |  | | 3.55 | | 4.32 | |  | | 3.38 | | 5.64 | |  | | 6.49 | 7.36 | |  | | 4.64 | | 9.61 | |  | 1.83 | | | 1.74 | |  | | 1.31 | | 1.77 | |
| G3 | | 0.48 | 0.51 |  | | 0.44 | | 0.56 | |  | | 3.35 | | 4.16 | |  | | 4.57 | | 4.43 | |  | | 6.49 | 7.70 | |  | | 6.77 | | 7.69 | |  | 1.94 | | | 1.85 | |  | | 1.49 | | 1.74 | |
| G4 | | 0.49 | 0.43 |  | | 0.49 | | 0.54 | |  | | 3.56 | | 3.56 | |  | | 3.79 | | 4.03 | |  | | 6.85 | 7.42 | |  | | 6.41 | | 7.16 | |  | 1.93 | | | 2.09 | |  | | 1.70 | | 1.78 | |
| G5 | | 0.38 | 0.47 |  | | 0.45 | | 0.59 | |  | | 4.03 | | 4.16 | |  | | 3.90 | | 4.87 | |  | | 6.98 | 7.28 | |  | | 7.36 | | 9.64 | |  | 1.74 | | | 1.75 | |  | | 1.89 | | 1.99 | |
| G6 | | 0.43 | 0.54 |  | | 0.45 | | 0.69 | |  | | 3.35 | | 3.67 | |  | | 4.17 | | 4.86 | |  | | 6.10 | 6.76 | |  | | 6.92 | | 7.88 | |  | 1.82 | | | 1.85 | |  | | 1.68 | | 1.62 | |
| G7 | | 0.39 | 0.42 |  | | 0.60 | | 0.55 | |  | | 3.82 | | 4.71 | |  | | 8.07 | | 9.82 | |  | | 6.29 | 6.76 | |  | | 8.87 | | 10.12 | |  | 1.66 | | | 1.43 | |  | | 1.10 | | 1.06 | |
| G8 | | 0.44 | 0.49 |  | | 0.43 | | 0.51 | |  | | 4.16 | | 4.16 | |  | | 4.43 | | 4.56 | |  | | 7.18 | 6.94 | |  | | 6.58 | | 8.92 | |  | 1.73 | | | 1.67 | |  | | 1.50 | | 1.95 | |
| G9 | | 0.55 | 0.57 |  | | 0.47 | | 0.63 | |  | | 3.90 | | 4.71 | |  | | 4.29 | | 3.78 | |  | | 6.48 | 8.55 | |  | | 7.89 | | 8.12 | |  | 1.66 | | | 1.82 | |  | | 1.85 | | 2.15 | |
| G10 | | 0.68 | 0.68 |  | | 0.44 | | 0.63 | |  | | 4.43 | | 4.73 | |  | | 3.79 | | 3.92 | |  | | 7.20 | 8.92 | |  | | 7.14 | | 8.43 | |  | 1.63 | | | 1.91 | |  | | 1.88 | | 2.17 | |
| G11 | | 0.61 | 0.56 |  | | 0.49 | | 0.73 | |  | | 4.29 | | 3.55 | |  | | 4.03 | | 4.03 | |  | | 7.63 | 7.58 | |  | | 7.88 | | 8.00 | |  | 1.78 | | | 2.13 | |  | | 1.96 | | 1.99 | |
| G12 | | 0.55 | 0.61 |  | | 0.54 | | 0.63 | |  | | 3.90 | | 4.29 | |  | | 3.90 | | 3.45 | |  | | 7.58 | 8.92 | |  | | 6.81 | | 7.65 | |  | 1.94 | | | 2.08 | |  | | 1.74 | | 2.22 | |
| G13 | | 0.39 | 0.45 |  | | 0.35 | | 0.49 | |  | | 3.15 | | 3.16 | |  | | 3.16 | | 3.16 | |  | | 5.89 | 6.33 | |  | | 5.86 | | 6.15 | |  | 1.87 | | | 2.00 | |  | | 1.86 | | 1.94 | |
| G14 | | 0.60 | 0.56 |  | | 0.43 | | 0.64 | |  | | 3.67 | | 3.45 | |  | | 3.78 | | 3.16 | |  | | 6.26 | 6.98 | |  | | 6.87 | | 6.73 | |  | 1.71 | | | 2.02 | |  | | 1.82 | | 2.13 | |
| G15 | | 0.56 | 0.41 |  | | 0.44 | | 0.65 | |  | | 2.90 | | 2.90 | |  | | 2.98 | | 2.98 | |  | | 6.57 | 6.25 | |  | | 7.25 | | 7.38 | |  | 2.26 | | | 2.14 | |  | | 2.44 | | 2.48 | |
| G16 | | 0.60 | 0.57 |  | | 0.43 | | 0.54 | |  | | 2.82 | | 2.99 | |  | | 2.98 | | 3.07 | |  | | 6.23 | 7.34 | |  | | 7.49 | | 7.45 | |  | 2.21 | | | 2.46 | |  | | 2.52 | | 2.43 | |
| G17 | | 0.61 | - |  | | 0.55 | | 0.57 | |  | | 2.99 | | - | |  | | 2.90 | | 2.75 | |  | | 6.66 | - | |  | | 6.44 | | 6.91 | |  | 2.23 | | | - | |  | | 2.22 | | 2.52 | |
| G18 | | 0.63 | 0.39 |  | | 0.38 | | 0.43 | |  | | 3.25 | | 3.07 | |  | | 3.47 | | 3.15 | |  | | 7.74 | 7.11 | |  | | 6.81 | | 7.32 | |  | 2.39 | | | 2.32 | |  | | 1.98 | | 2.32 | |
| G19 | | 0.51 | 0.49 |  | | 0.50 | | 0.69 | |  | | 3.07 | | 3.67 | |  | | 3.72 | | 3.90 | |  | | 6.69 | 8.55 | |  | | 7.19 | | 8.96 | |  | 2.18 | | | 2.33 | |  | | 1.94 | | 2.30 | |
| G20 | | 0.46 | 0.56 |  | | 0.55 | | 0.54 | |  | | 3.78 | | 3.90 | |  | | 4.86 | | 3.79 | |  | | 6.93 | 9.29 | |  | | 7.45 | | 7.45 | |  | 1.83 | | | 2.40 | |  | | 1.53 | | 1.97 | |
| G21 | | 0.49 | 0.69 |  | | 0.59 | | 0.55 | |  | | 3.45 | | 3.55 | |  | | 3.67 | | 4.03 | |  | | 7.34 | 8.45 | |  | | 8.36 | | 8.35 | |  | 2.13 | | | 2.38 | |  | | 2.28 | | 2.07 | |
| G22 | | 0.46 | 0.55 |  | | 0.53 | | 0.55 | |  | | 2.98 | | 3.25 | |  | | 3.15 | | 3.35 | |  | | 5.92 | 6.87 | |  | | 6.68 | | 7.18 | |  | 1.99 | | | 2.12 | |  | | 2.12 | | 2.14 | |
| G23 | | 0.66 | 0.74 |  | | 0.56 | | 0.62 | |  | | 3.07 | | 3.07 | |  | | 2.83 | | 2.82 | |  | | 6.12 | 7.77 | |  | | 5.92 | | 6.26 | |  | 2.00 | | | 2.53 | |  | | 2.10 | | 2.22 | |
| G24 | | 0.53 | 0.61 |  | | 0.56 | | 0.74 | |  | | 4.56 | | 3.67 | |  | | 5.51 | | 3.56 | |  | | 6.74 | 6.81 | |  | | 6.22 | | 6.85 | |  | 1.48 | | | 1.85 | |  | | 1.13 | | 1.93 | |
| G25 | | 0.62 | - |  | | 0.77 | | 0.80 | |  | | 4.43 | | - | |  | | 4.43 | | 3.67 | |  | | 6.51 | - | |  | | 6.51 | | 7.53 | |  | 1.47 | | | - | |  | | 1.48 | | 2.06 | |
| G26 | | 0.60 | 0.52 |  | | 0.53 | | 0.56 | |  | | 3.47 | | 3.90 | |  | | 3.78 | | 6.99 | |  | | 6.56 | 8.36 | |  | | 7.27 | | 10.31 | |  | 1.90 | | | 2.14 | |  | | 1.92 | | 1.47 | |
| G27 | | 0.56 | 0.50 |  | | 0.47 | | 0.50 | |  | | 3.16 | | 3.35 | |  | | 4.03 | | 4.16 | |  | | 7.45 | 7.05 | |  | | 6.95 | | 7.18 | |  | 2.36 | | | 2.10 | |  | | 1.74 | | 1.73 | |
| G28 | | 0.46 | 0.51 |  | | 0.46 | | 0.51 | |  | | 2.98 | | 2.99 | |  | | 3.15 | | 3.25 | |  | | 6.45 | 6.30 | |  | | 6.57 | | 9.05 | |  | 2.17 | | | 2.12 | |  | | 2.08 | | 2.79 | |
| G29 | | 0.46 | 0.59 |  | | 0.60 | | 0.80 | |  | | 2.98 | | 3.50 | |  | | 3.07 | | 3.07 | |  | | 7.09 | 7.70 | |  | | 6.42 | | 7.49 | |  | 2.38 | | | 2.24 | |  | | 2.09 | | 2.45 | |
| G30 | | 0.69 | 0.55 |  | | 0.76 | | 0.66 | |  | | 3.15 | | 3.55 | |  | | 3.07 | | 3.01 | |  | | 7.72 | 7.35 | |  | | 6.75 | | 7.26 | |  | 2.45 | | | 2.07 | |  | | 2.19 | | 2.42 | |
| G31 | | 0.44 | 0.45 |  | | - | | 0.51 | |  | | 5.17 | | 4.57 | |  | | - | | 3.67 | |  | | 7.72 | 8.39 | |  | | - | | 8.05 | |  | 1.49 | | | 1.83 | |  | | - | | 2.20 | |
| G32 | | 0.42 | 0.49 |  | | - | | 0.51 | |  | | 3.78 | | 3.72 | |  | | - | | 4.44 | |  | | 7.26 | 8.67 | |  | | - | | 8.04 | |  | 1.92 | | | 2.38 | |  | | - | | 1.82 | |
| G33 | | 0.40 | 0.65 |  | | - | | 0.55 | |  | | 3.35 | | 3.90 | |  | | - | | 3.35 | |  | | 6.45 | 9.28 | |  | | - | | 7.35 | |  | 1.93 | | | 2.38 | |  | | - | | 2.20 | |
| G34 | | 0.48 | 0.42 |  | | - | | 0.57 | |  | | 3.35 | | 2.98 | |  | | - | | 2.99 | |  | | 6.08 | 5.75 | |  | | - | | 7.55 | |  | 1.82 | | | 1.93 | |  | | - | | 2.55 | |
| G35 | | 0.50 | 0.59 |  | | - | | 0.48 | |  | | 2.98 | | 2.83 | |  | | - | | 3.27 | |  | | 6.12 | 6.06 | |  | | - | | 6.93 | |  | 2.05 | | | 2.15 | |  | | - | | 2.11 | |
| G36 | | 0.36 | 0.47 |  | | 0.57 | | 0.49 | |  | | 3.78 | | 5.02 | |  | | 3.45 | | 3.90 | |  | | 6.00 | 8.62 | |  | | 6.97 | | 7.49 | |  | 1.59 | | | 1.72 | |  | | 2.02 | | 1.92 | |
| LSD_E_(α = 0.05) | | 0.18 | 0.18 |  | | 0.17 | | 0.21 | |  | | 0.50 | | 0.63 | |  | | 0.59 | | 1.06 | |  | | 0.85 | 1.65 | |  | | 1.81 | | 1.25 | |  | 0.27 | | | 0.54 | |  | | 0.47 | | 0.35 | |
| LSD_E×Y_ (α=0.05) | | 0.17 | |  | | 0.19 | | | |  | | 0.56 | | | |  | | 0.86 | | | |  | | 1.31 | | |  | | 1.51 | | | |  | 0.43 | | | | |  | | 0.40 | | | |

| **S1 Table -** Continued | | | | | | | | | | | | | | | | | | | | | | | | |
| --- | --- | --- | --- | --- | --- | --- | --- | --- | --- | --- | --- | --- | --- | --- | --- | --- | --- | --- | --- | --- | --- | --- | --- | --- |
| Traits | Water SRC (%) | | | | | |  | Sucrose SRC (%) | | | | |  | Lactic acid SRC (%) | | | | |  | Sodium carbonate SRC (%) | | | | |
| Genotype | 2019 | |  | 2020 | | |  | 2019 | |  | 2020 | |  | 2019 | |  | 2020 | |  | 2019 | |  | 2020 | |
|  | WW | WS |  | WW | | WS |  | WW | WS |  | WW | WS |  | WW | WS |  | WW | WS |  | WW | WS |  | WW | WS |
| G1 | 118.68 | 105.14 |  | 113.23 | | 134.94 |  | 115.30 | 147.74 |  | 141.95 | 163.75 |  | 160.04 | 119.14 |  | 163.82 | 144.82 |  | 119.02 | 121.70 |  | 134.18 | 132.45 |
| G2 | 102.31 | 104.20 |  | 97.95 | | 135.29 |  | 122.32 | 143.66 |  | 140.28 | 165.00 |  | 133.34 | 124.11 |  | 163.78 | 145.34 |  | 107.48 | 124.91 |  | 124.70 | 134.99 |
| G3 | 96.98 | 114.89 |  | 103.61 | | 128.48 |  | 130.32 | 163.39 |  | 146.82 | 159.06 |  | 136.29 | 129.81 |  | 175.30 | 145.00 |  | 125.60 | 146.66 |  | 124.64 | 137.92 |
| G4 | 102.67 | 107.30 |  | 96.37 | | 114.77 |  | 130.40 | 151.97 |  | 129.67 | 153.23 |  | 136.26 | 118.67 |  | 156.96 | 134.43 |  | 114.08 | 128.46 |  | 107.46 | 127.16 |
| G5 | 110.43 | 116.95 |  | 101.47 | | 146.13 |  | 130.36 | 147.11 |  | 136.99 | 177.29 |  | 153.66 | 127.82 |  | 167.20 | 163.28 |  | 119.81 | 132.77 |  | 109.44 | 154.40 |
| G6 | 109.16 | 122.51 |  | 99.93 | | 124.58 |  | 130.62 | 155.91 |  | 146.97 | 155.48 |  | 144.76 | 132.01 |  | 161.69 | 132.76 |  | 123.58 | 131.55 |  | 113.60 | 141.29 |
| G7 | 115.81 | 123.62 |  | 145.07 | | 163.67 |  | 130.18 | 161.12 |  | 186.82 | 189.66 |  | 169.19 | 144.91 |  | 203.05 | 181.17 |  | 134.57 | 148.18 |  | 161.86 | 163.36 |
| G8 | 117.13 | 111.35 |  | 108.00 | | 121.27 |  | 145.26 | 144.31 |  | 149.10 | 160.66 |  | 156.90 | 125.68 |  | 172.50 | 159.14 |  | 135.40 | 127.76 |  | 128.40 | 144.09 |
| G9 | 91.41 | 101.10 |  | 93.53 | | 96.76 |  | 115.51 | 132.95 |  | 128.02 | 141.77 |  | 133.16 | 111.53 |  | 152.12 | 109.97 |  | 129.91 | 147.64 |  | 125.04 | 134.76 |
| G10 | 94.43 | 100.46 |  | 89.53 | | 97.82 |  | 117.00 | 138.05 |  | 127.28 | 130.43 |  | 129.41 | 118.32 |  | 135.48 | 108.99 |  | 125.73 | 136.06 |  | 121.27 | 128.16 |
| G11 | 87.09 | 94.50 |  | 91.16 | | 97.39 |  | 116.61 | 129.00 |  | 134.20 | 136.73 |  | 134.70 | 105.22 |  | 146.72 | 107.15 |  | 120.74 | 138.00 |  | 129.47 | 133.98 |
| G12 | 88.04 | 97.27 |  | 88.57 | | 101.14 |  | 116.19 | 126.64 |  | 131.16 | 138.94 |  | 140.51 | 115.24 |  | 146.54 | 128.05 |  | 122.71 | 128.56 |  | 126.45 | 121.44 |
| G13 | 93.72 | 103.81 |  | 90.77 | | 103.15 |  | 115.00 | 141.33 |  | 124.89 | 127.34 |  | 121.42 | 92.48 |  | 111.43 | 88.05 |  | 108.51 | 118.73 |  | 104.29 | 115.91 |
| G14 | 86.43 | 90.74 |  | 83.88 | | 96.54 |  | 113.14 | 119.05 |  | 125.67 | 126.96 |  | 128.95 | 100.02 |  | 122.67 | 104.51 |  | 108.13 | 111.99 |  | 111.65 | 101.22 |
| G15 | 94.66 | 98.83 |  | 89.79 | | 96.99 |  | 120.35 | 129.36 |  | 121.07 | 130.32 |  | 132.04 | 101.24 |  | 117.47 | 98.51 |  | 110.62 | 109.09 |  | 106.87 | 108.61 |
| G16 | 82.40 | 96.92 |  | 87.54 | | 102.57 |  | 117.10 | 127.17 |  | 117.12 | 129.21 |  | 118.74 | 94.07 |  | 118.79 | 92.11 |  | 114.37 | 140.32 |  | 110.65 | 134.17 |
| G17 | 86.90 | - |  | 88.07 | | 101.83 |  | 112.94 | - |  | 121.15 | 130.44 |  | 122.94 | - |  | 114.69 | 90.68 |  | 109.18 | - |  | 109.33 | 132.16 |
| G18 | 90.10 | 107.44 |  | 100.80 | | 112.78 |  | 125.34 | 137.94 |  | 124.35 | 154.23 |  | 133.54 | 113.34 |  | 130.29 | 139.08 |  | 109.89 | 131.47 |  | 102.29 | 138.37 |
| G19 | 93.15 | 111.88 |  | 105.62 | | 121.00 |  | 131.92 | 151.97 |  | 133.63 | 160.19 |  | 134.24 | 122.32 |  | 152.04 | 153.18 |  | 101.74 | 148.15 |  | 119.47 | 147.24 |
| G20 | 89.01 | 111.84 |  | 96.93 | | 109.05 |  | 122.19 | 146.87 |  | 127.28 | 152.23 |  | 133.23 | 114.52 |  | 145.65 | 134.39 |  | 104.27 | 142.64 |  | 117.92 | 124.63 |
| G21 | 104.36 | 108.06 |  | 114.49 | | 143.81 |  | 142.02 | 148.19 |  | 168.16 | 168.45 |  | 147.84 | 103.81 |  | 187.33 | 167.53 |  | 127.34 | 143.69 |  | 137.84 | 141.46 |
| G22 | 89.72 | 100.19 |  | 129.76 | | 104.80 |  | 120.22 | 138.62 |  | 147.38 | 148.36 |  | 127.92 | 120.23 |  | 162.05 | 135.22 |  | 114.88 | 122.90 |  | 121.22 | 131.80 |
| G23 | 82.30 | 97.93 |  | 87.35 | | 101.23 |  | 118.36 | 131.67 |  | 119.61 | 131.24 |  | 125.72 | 99.26 |  | 111.20 | 100.55 |  | 121.49 | 136.89 |  | 100.36 | 120.45 |
| G24 | 106.26 | 109.29 |  | 146.02 | | 131.18 |  | 134.09 | 139.20 |  | 168.62 | 171.82 |  | 143.49 | 126.47 |  | 189.52 | 145.89 |  | 146.44 | 160.04 |  | 153.25 | 158.88 |
| G25 | 89.93 | - |  | 112.50 | | 107.70 |  | 118.42 | - |  | 150.66 | 152.00 |  | 125.76 | - |  | 168.88 | 120.31 |  | 118.93 | - |  | 131.50 | 137.87 |
| G26 | 87.62 | 110.43 |  | 107.59 | | 181.00 |  | 124.55 | 152.42 |  | 147.63 | 230.23 |  | 131.73 | 124.81 |  | 165.70 | 193.06 |  | 129.42 | 159.07 |  | 127.69 | 187.17 |
| G27 | 110.50 | 120.49 |  | 111.78 | | 113.69 |  | 143.46 | 159.13 |  | 150.61 | 166.65 |  | 156.44 | 130.37 |  | 155.08 | 151.25 |  | 131.01 | 148.97 |  | 124.71 | 142.57 |
| G28 | 90.42 | 104.08 |  | 95.78 | | 145.42 |  | 126.73 | 139.48 |  | 127.38 | 176.31 |  | 128.62 | 100.44 |  | 128.64 | 150.84 |  | 116.31 | 132.11 |  | 114.74 | 156.16 |
| G29 | 86.43 | 101.82 |  | 90.68 | | 109.71 |  | 124.92 | 141.42 |  | 130.10 | 153.30 |  | 129.98 | 115.03 |  | 130.29 | 130.38 |  | 109.43 | 135.10 |  | 114.18 | 134.23 |
| G30 | 92.36 | 113.79 |  | 100.98 | | 114.41 |  | 132.76 | 148.28 |  | 135.00 | 157.59 |  | 128.24 | 122.98 |  | 136.86 | 126.41 |  | 124.89 | 165.49 |  | 123.72 | 141.54 |
| G31 | 102.48 | 114.13 |  | - | | 137.65 |  | 147.40 | 154.70 |  | - | 145.46 |  | 152.15 | 132.76 |  | - | 137.84 |  | 148.06 | 164.66 |  | - | 163.91 |
| G32 | 82.35 | 113.39 |  | - | | 122.32 |  | 118.95 | 147.28 |  | - | 153.16 |  | 126.15 | 125.46 |  | - | 135.77 |  | 113.45 | 159.88 |  | - | 139.36 |
| G33 | 88.57 | 116.58 |  | - | | 111.78 |  | 116.61 | 158.30 |  | - | 140.31 |  | 123.09 | 122.41 |  | - | 100.73 |  | 118.30 | 145.67 |  | - | 127.79 |
| G34 | 86.94 | 92.16 |  | - | | 102.96 |  | 112.83 | 122.92 |  | - | 134.28 |  | 116.55 | 76.29 |  | - | 92.54 |  | 105.68 | 109.97 |  | - | 122.77 |
| G35 | 94.63 | 110.19 |  | - | | 131.67 |  | 118.52 | 137.24 |  | - | 177.49 |  | 126.99 | 97.87 |  | - | 123.61 |  | 116.10 | 128.56 |  | - | 141.02 |
| G36 | 98.96 | 134.48 |  | 93.53 | | 141.68 |  | 133.63 | 171.95 |  | 140.71 | 159.28 |  | 162.64 | 150.52 |  | 144.19 | 136.64 |  | 123.16 | 151.26 |  | 122.28 | 136.08 |
| LSD_E_(α = 0.05) | 6.04 | 7.96 |  | | 6.86 | 9.49 |  | 8.70 | 11.25 |  | 8.60 | 12.41 |  | 10.13 | 11.85 |  | 11.61 | 10.86 |  | 9.21 | 13.25 |  | 11.43 | 9.86 |
| LSD_E×Y_ (α=0.05) | 6.91 | |  | 8.23 | | |  | 9.83 | |  | 10.62 | |  | 10.80 | |  | 11.00 | |  | 11.14 | |  | 10.41 | |
